# Amorphous Carbon Coatings for Total Knee Replacements—Part I: Deposition, Cytocompatibility, Chemical and Mechanical Properties

**DOI:** 10.3390/polym13121952

**Published:** 2021-06-11

**Authors:** Benedict Rothammer, Kevin Neusser, Max Marian, Marcel Bartz, Sebastian Krauß, Thomas Böhm, Simon Thiele, Benoit Merle, Rainer Detsch, Sandro Wartzack

**Affiliations:** 1 Engineering Design, Friedrich-Alexander-University Erlangen-Nuremberg (FAU), Martensstr. 9, 91058 Erlangen, Germany; kevin.k.neusser@fau.de (K.N.); marian@mfk.fau.de (M.M.); bartz@mfk.fau.de (M.B.); wartzack@mfk.fau.de (S.W.); 2 Materials Science & Engineering, Institute I, Interdisciplinary Center for Nanostructured Films (IZNF), Friedrich-Alexander-University Erlangen-Nuremberg (FAU), Cauerstr. 3, 91058 Erlangen, Germany; sebastian.s.krauss@fau.de (S.K.); benoit.merle@fau.de (B.M.); 3 Forschungszentrum Jülich GmbH, Helmholtz-Institute Erlangen-Nürnberg for Renewable Energy, Cauerstr. 1, 91058 Erlangen, Germany; t.boehm@fz-juelich.de (T.B.); si.thiele@fz-juelich.de (S.T.); 4 Department of Chemical and Biological Engineering, Friedrich-Alexander-University Erlangen-Nuremberg (FAU), Egerlandstr. 3, 91058 Erlangen, Germany; 5 Department of Materials Science and Engineering, Institute of Biomaterials, Friedrich-Alexander-University Erlangen-Nuremberg (FAU), Cauerstr. 6, 91058 Erlangen, Germany; rainer.detsch@fau.de

**Keywords:** DLC coating, biomedical applications, biotribology, UHMWPE, CoCr, Ti64, total knee arthroplasty, cytocompatibility, hardness, adhesion

## Abstract

Diamond-like carbon (DLC) coatings have the potential to reduce implant wear and thus to contribute to avoiding premature failure and increase service life of total knee replacements (TKAs). This two-part study addresses the development of such coatings for ultrahigh molecular weight polyethylene (UHMWPE) tibial inlays as well as cobalt–chromium–molybdenum (CoCr) and titanium (Ti64) alloy femoral components. While a detailed characterization of the tribological behavior is the subject of part II, part I focusses on the deposition of pure (a-C:H) and tungsten-doped hydrogen-containing amorphous carbon coatings (a-C:H:W) and the detailed characterization of their chemical, cytological, mechanical and adhesion behavior. The coatings are fabricated by physical vapor deposition (PVD) and display typical DLC morphology and composition, as verified by focused ion beam scanning electron microscopy and Raman spectroscopy. Their roughness is higher than that of the plain substrates. Initial screening with contact angle and surface tension as well as in vitro testing by indirect and direct application indicate favorable cytocompatibility. The DLC coatings feature excellent mechanical properties with a substantial enhancement of indentation hardness and elastic modulus ratios. The adhesion of the coatings as determined in modified scratch tests can be considered as sufficient for the use in TKAs.

## 1. Introduction

The failure of the natural human knee joint due to gonarthrosis or rheumatoid arthritis requires the implantation of a total knee arthroplasty (TKA) in order to restore joint functionality and provide patients with a pain-free and more mobile life [1]. Thereby, the number of primary TKA surgeries worldwide is continuously increasing, especially in the group of younger patients [2,3,4]. Generally, TKAs consist of ultra-high molecular weight (UHWMPE) or highly cross-linked polyethylene (HXLPE) tibial plateaus as well as cobalt-chromium-molybdenum alloy (CoCr), titanium alloy (Ti64) or oxide ceramic femoral components. CoCr is currently dominating due to the more favorable tribological behavior compared to Ti64 though the latter being considered more biocompatible [5]. Although the service life of most TKAs can reach up to 25 years [6,7], postsurgical infections and aseptic loosening of endoprostheses due to multiple etiologies can lead to premature failure [8]. Wear particles from the implant materials are believed to be major causes of aseptic endoprosthesis loosening [9]. In addition to optimizing the TKA type [5,10], wear-resistant material pairings or surface modifications are pursued [11,12]. The application of biotribologically effective coatings on the articulating implant surfaces is a particularly promising approach to increase wear resistance [13,14]. In fact, the excellent properties of metallic and polymeric implant materials, including high ductility and damage-tolerance, can be combined with the advantages of ceramic materials, such as high hardness and chemical inertness [5,13], saving patients from undergoing revision surgery [9,12]. Due to the interaction with human tissue and synovial fluid with their large variety of cells and proteins, the coatings require sufficiently high biocompatibility [12] and adhesion. Repenning [15] summarized the aim of biotribologically effective coatings as a biological, chemical and physical modification of the interface between implant and tissue in order to enable a continuous improvement of functionality with respect to the biological environment.

Several coatings for possible biotribological application on TKAs can be efficiently fabricated by physical (PVD) or plasma enhanced chemical vapor deposition (PECVD) [16]. These can be classified into metallic [17], nitride [18,19,20], oxide [21,22] or amorphous/diamond-like carbon (DLC) coatings [23,24]. The application of DLC coatings is considered to be particularly suitable for wear reduction due to the advantageous ratio of hardness to elasticity in combination with the ability to form transfer films. Moreover, the outstanding mechanical properties [23,25], which are tunable over a wide range, can be supplemented by medically relevant properties [26]. These include chemical resistance, antibacterial behavior [27], and good biocompatibility [28]. The tribologically advantageous behavior particularly originates from hydrogen release, which results in lattice relaxation, subsequent shear deformation and the formation of graphitic surfaces [12]. This velocity and load dependent process is called wear-induced surface graphitization [26] and leads to the formation of wear-protective and friction-reducing transfer films to the counterbody. Friction and wear behavior as well as surface tension and residual stresses can be further tailored by modifying the coating with doping elements. Typically, metallic doping elements, such as titanium (Ti), copper (Cu), tungsten (W), niobium (Nb), gold (Au), silver (Ag), chromium (Cr), or tantalum (Ta), are used to enhance the ductility of DLC coatings [29]. The surface tension can be influenced by non-metallic doping elements, e.g., nitrogen (N), silicon (Si), oxygen (O), fluorine (F), or phosphorus (P) [29]. Furthermore, P-doped DLC coatings reduce the interaction of the coating with plasma proteins, Si-doping lowers platelet attachment and improves endothelial cell attachment, and F-doping suppresses platelet activation [28]. Protein adsorption [12] and cell growth of tissue-forming cells can be influenced by doping with Ti [30] or calcium oxide (CaO) [24]. However, the basic requirement for wear resistance and biocompatibility is a sufficient coating adhesion to the implant substrates [23,25,26].

The adhesion of the DLC coating to metallic substrates can be improved by ion etching of the substrate surface prior to the actual coating process, since this removes the passivating oxide layers [31]. As DLC coatings exhibit intrinsic residual stresses induced by the high-energy coating processes [32], the coating thickness influences to a large extend the adhesion and the tendency to spallation or delamination as well as the formation of wear particles [33]. Increasing the hydrogen content in the DLC layer can also lessen residual stresses and promote adhesion but reduces hardness. Therefore, multilayer coating architectures or intermediate layers are frequently employed [34,35]. To ensure good coating adhesion on Ti64 and CoCr, Ti- [36] and Cr-based [37] metal-carbide [26] or metal-nitride [38,39] interlayers have been used. A gradient from the intermediate into the desired DLC layer avoids carbon accumulation at the interfaces and increases cohesion [38]. Also, W- [40], Ag- [41], or Ti-doping [42] of the DLC coating reduces diamond bonding contents, favoring ductility and adhesion. Tungsten-containing amorphous carbon coatings [40] are particularly promising due to their good mechanical properties [43] and medical compatibility [44].

With regard to increasing the service life of TKAs, special emphasis has to be put on protecting polymeric tibial inserts from wear [45]. Owing to their chemical similarity to UHMWPE, hydrogenated amorphous carbon coatings are specially well suited for this purpose [46]. After argon- (Ar) or N-ion plasma etching, they can be directly deposited on the substrate by PVD or PECVD [39,46]. Naturally, this involves the risk of spontaneous coating delamination due to large differences in hardness and stiffness between the coating and the substrate, leading to increased wear debris [43] and tribologically induced undesirable cell biological interactions [26]. However, it was shown by He et al. [47] that such coating structures can achieve acceptable adhesion. Similarly to metallic substrates, doping elements such as Ag, N, or Si can be employed to adjust adhesion, medically relevant properties or the tribological behavior [17,27,48,49].

To summarize, DLC coatings on metallic and polymeric substrates have the potential to reduce wear and therefore to boost the service life of joint replacements. To this end, coating deposition and composition, the surface topography, cytocompatibility, chemical and mechanical properties, as well as the adhesion to the substrate, have to be aligned. Consequently, this two-part study focuses on the development of wear reducing DLC coating systems specifically for Ti64 and CoCr femoral as well as UHMWPE tibial components of TKAs. While the biotribological behavior is investigated in part II [50] using pin-on-disk model tests as well as a detailed wear analysis, this part I addresses the coating deposition and static characterization. The aim is to evaluate the in vitro behavior, mechanical and adhesive properties and to discuss the applicability to the articulating surfaces of knee joint replacements. To this end, the PVD-fabricated amorphous carbon (DLC) coatings were characterized by roughness and film thickness measurements, focused ion beam scanning electron microscopy (FIB-SEM), Raman spectroscopy, contact angle measurements, indirect and direct cell testing, indentation hardness and elasticity measurements as well as Rockwell and scratch tests.

## 2. Materials and Methods

### 2.1. Materials

The investigated substrate materials were UHMWPE [51] (Chirulen^®^ GUR 1020, Quadrant EPP, Vreden, Germany), Co28Cr6Mo [52] (CoCr, Peter Brehm, Weisendorf, Germany) and Ti6Al4V ELI [53] (Ti64, Jäckel + Co. Edelstahl Metalltechnik, Schöneck, Germany) with medical standards. The geometries of the samples to be coated encompassed both flat specimens for chemical, mechanical and cytological characterization, as well as disks and pins for tribometer tests (see [50]). The UHMWPE disks had a diameter of 45 mm and a height of 8 mm while the CoCr and Ti64 disks had a diameter of 25 mm and a height of 5 mm. The CoCr and Ti64 pins were 10 mm in diameter, 30 mm in length, and had a head radius of 100 mm. The specimens were mirror-polished (Saphir 550—Rubin 520, ATM Qness, Mammelzen, Germany) and ultrasonically cleaned (Sonorex Super RK 255H 160 W 35 Hz, Bandelin electronic, Berlin, Germany) in acetone (except UHMWPE) and isopropyl alcohol.

### 2.2. Coating Deposition

**Figure 1:**
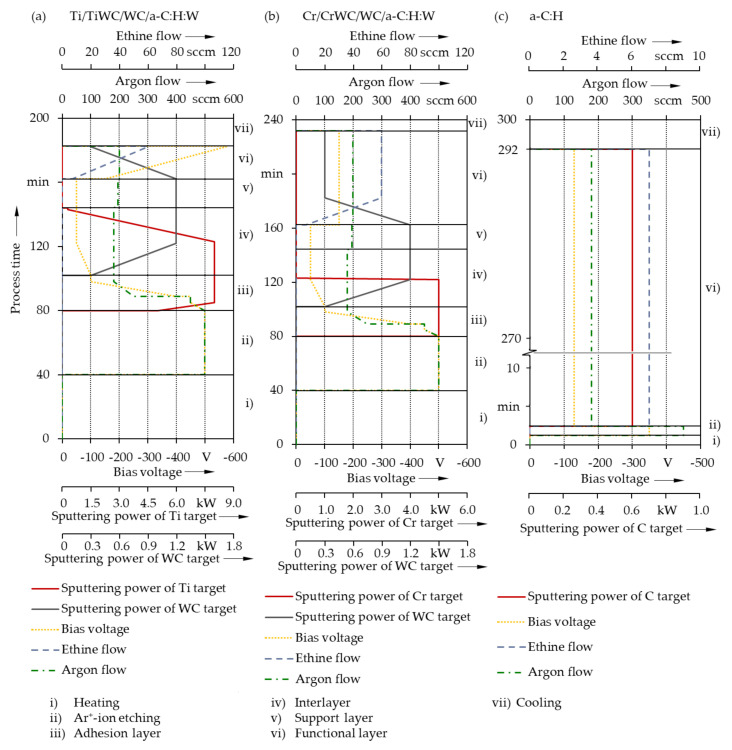
Main deposition process parameters as a function of time for Ti/TiWC/WC/a-C:H:W on Ti64 (**a**), Cr/CrWC/WC/a-C:H:W on CoCr (**b**), and a-C:H on UHMWPE (**c**).

Under twofold rotation, Cr/CrWC/WC/a-C:H:W and Ti/TiWC/WC/a-C:H:W coating systems were deposited on CoCr and Ti64 and a single-layer a-C:H coating was fabricated on UHMWPE using an industrial-scale coating plant (TT 300 K4, H-O-T Härte- und Oberflächentechnik, Nuremberg, Germany) for physical vapor deposition and plasma-enhanced chemical vapor deposition (PVD/PECVD). Prior to the actual deposition, the chamber was evacuated to a base pressure of at least 5.0 × 10^−4^ Pa. Subsequently, in case of metallic substrates, the recipient was heated to 300 °C for 40 min and then the specimens were cleaned and activated for 40 min in argon^+^-ion plasma with a bipolar pulsed bias voltage of −500 V and an argon flow of 500 sccm. The chamber was not heated prior to deposition onto UHMWPE to minimize deposition-related heat flux into UHMWPE. However, UHMWPE was also cleaned and activated using argon^+^-ion plasma etching with a bipolar pulsed bias voltage of −350 V and an argon flow of 450 sccm for 2 min. Particularly for the metallic substrates, care was taken to ensure graded interfaces on the substrate and between the single layers through a smooth variation of the coating process parameters, as illustrated in Figure 1.

With the Ti64 and CoCr substrates, a thin adhesion layer of Ti, respectively Cr was first deposited. It was complemented by a further interlayer of tungsten carbide (WC), which boosted the adhesion to the metallic substrates. The top, biotribologically effective functional layers, consisted of tungsten-containing hydrogenated amorphous carbon (a-C:H:W). In contrast, the biotribologically effective a-C:H functional layer was directly applied on UHMWPE due to the chemical similarity of substrate and coating [46]. The deposition time was set to obtain a thickness of roughly 1.0 µm for the a-C:H:W and 1.5 μm the a-C:H coating. Both layers were deposited by reactive PVD through medium frequency (MF) unbalanced magnetron (UBM) sputtering from binder-free WC and graphite (C, purity 99.998%) targets under argon–ethine (Ar–C_2_H_2_) atmosphere (Ar purity 99.999%, C_2_H_2_ purity 99.5%). The Cr and Ti adhesion layers as well as the CrWC and TiWC interlayers were fabricated by sputtering from a powder metallurgical Cr (purity 99.9%), a melting metallurgical Ti (purity 99.6%) as well as WC (purity 99.9%) targets (dimensions 170 × 267.5 mm) with bipolar pulsed voltages. The voltage setpoints corresponded to the negative pulse amplitudes, while the positive pulses were expressed by 15% of the voltage setpoints. A pulse frequency *f* of 40 kHz with a reverse recovery time *RRT* of 5 μs was set for the Cr, Ti, WC targets. The deposition of the a-C:H layer required a slight adjustment of the pulse parameters to 75 kHz and 3 μs to ensure stable and efficient cathode operation. A negative direct current (DC) bias voltage was used for all actual deposition processes. The process temperature during the deposition of the a-C:H:W functional layers on the metallic substrates was thus kept between 100 °C and 135 °C and the a-C:H functional layer on UHMWPE below 50 °C.

### 2.3. Morphology and Structure Characterization

For the determination of the surfaces’ micro-geometry, the relevant roughness parameters were measured tactilely (Form Talysurf^®^ PGI Novus E15, Taylor Hobson, Leicester, UK) according to DIN EN ISO 4287 [54], 4288 [55], 13565-1 [56] and 13565-2 [57]. Mean values were calculated from five measurements with an angular offset of 72° on each specimen. The coating thicknesses were determined using the crater-grinding method according to DIN EN ISO 26423 [58]. Therefore, five spherical grindings were generated (KSG-2, KTmfk, Erlangen, Germany) and measured with optical light microscopy (DM4000 M and Leica Application Suite V4.9, Leica Microsystems, Wetzlar, Germany). The morphology and structure of the coatings were further characterized by FIB-SEM (Helios NanoLab 600i, FEI Thermo Fisher, Hillsboro, OR, USA) with an acceleration voltage of 5 kV, an electron current of 0.69 nA and working distances between 2.0 and 4.1 mm. Imaging of the cross-sections was carried out at a tilting angle of 52°. Respective results are shown and discussed in Section 3.1.

### 2.4. Chemical and Cytological Characterization

Raman spectra were acquired with excitation at 457 nm with 0.15 mW laser power (WITec alpha300, WITec, Ulm, Germany), whereby each spectrum was integrated for 2 s with 5 accumulations. The spectra were background-corrected using the shape-based algorithm in WITec Project FIVE+. After normalizing the spectra to maximum signal intensity, mean spectra from different spots on specimens were obtained.

Furthermore, contact angle measurements according to DIN EN 828 [59] were used to calculate the surface energies by utilizing the sessile drop method. Thereby, the drop was placed with a dispensing syringe and imaged using a CCD camera with x2.5 magnification (DSA10, Krüss, Hamburg, Germany). The drop profile and contact angle to the base line were determined using the Laplace–Young method. Thereby, distilled water, ethylene glycol and diiodomethane with different disperse and polar contributions were used as testing liquids to calculate the surface free energy according to the OWRK theory for coatings [60,61,62]. Five measurements per liquid were taken at different locations of each specimen at an ambient temperature of 20 °C.

Biocompatibility is a complex material property, which can be determined by *in vitro*, in vivo and clinical tests [63]. The in vitro biocompatibility evaluation (cytocompatibility testing) of materials or eluates is the first step in the assessment of biological behavior of biomedical devices and implants [64]. Prior to eluate preparation, the specimens were sterilized in an autoclave (D-45, Systec, Linden, Germany) at a temperature of 121 °C and for a duration of 20 min. Subsequently, the eluates were prepared by incubating at a temperature of 37 °C for 24 h and put individually into vessels containing culture media. The released substances from the specimens formed the extraction medium (indirect cell test). The human osteoblast-like cell line MG-63 (Sigma-Aldrich, Germany) was used for the cell experiments at a temperature of 37 °C in a water vapor-saturated, 5% CO_2_ air atmosphere. Dulbecco’s Modified Eagle Medium (DMEM) was used as culture medium and enriched with 10% fetal calf serum (FCS) and 1% penicillin/streptomycin. For the in vitro investigations, 96-well plates were used with an inoculum of 10,000 cells MG-63 cells per 0.1 mL medium and well. These cell cultures were pre-cultured for 24 h. Subsequently, the cell culture media were replaced by sample eluates (0.1 mL) and the cells were cultured again for 24 h therein. For the cell viability analysis, a master matrix prepared from 4 μL calcein per 1 mL Phosphate-buffered saline (PBS) was used to visualize the live cells after incubation with eluates. After removal of the eluate, the calcein master mix was pipetted into the wells and incubated for 45 min. Next, the stained cells were fixed by incubation with a solution of formaldehyde (concentration 3.7%) in PBS for 15 min. During this process, the cells died but remained fixed in their initial form. 4′,6-diamidino-2-phenylindole (DAPI) was used to stain the nuclei, and 1 μL of DAPI was added to each specimen per 1 mL of PBS. After 5 min of incubation, the nuclei of dead cells were stained by this procedure due to their disrupted membranes. The final step was to pipette off the DAPI/PBS solution and add pure PBS. Cells were analyzed using a transmission microscope with fluorescent light at ×100 and ×200 magnification (Observer, Zeiss, Oberkochen, Germany). Based on these images, the cell number was determined and compared by counting the nuclei using Image J 1.53 software. The difference to untreated (positive) controls was directly proportional to a possible cytotoxic effect of the eluates.

Furthermore, the specimens were examined for direct seeding of cells on the specimens (direct cell test) in order to analyze the cell-material interaction. The test performance was based upon the same steps and equipment as described for the indirect tests. Three replicates per specimen were prepared and analyzed. While a 12-well plate was used for the metallic specimens, a 6-well plate was used for the UHMWPE specimens. Each well was filled with 1 mL (Ti64, CoCr) or 7 mL (UHMWPE) medium, resulting in roughly 100,000 cells per well, and the cell density on the specimens was evaluated in this test. Respective results are shown and discussed in Section 3.2.

### 2.5. Mechanical Characterization

The indentation hardness *H*_IT_ and indentation modulus *E*_IT_ were determined by nanoindentation with Vickers tips (Picodentor HM500 and WinHCU, Helmut Fischer, Sindelfingen, Germany) following [65,66] while ensuring that the maximum indentation depth was substantially less than 10% of the coating thicknesses to minimize substrate influences [67,68]. Also with respect to the surface roughness, lower forces were found to be suitable to deliver reproducible results. Besides the coatings, the substrates were examined as references using the same methodology as well. For statistical purposes, 10 indentations per specimen with respective interdistances higher than 40 µm were performed and evaluated. The elastic-plastic parameters were calculated assuming typical values for the Poisson’s ratios for amorphous carbon coatings as well as the substrates [69,70,71]. The respective parameters and settings for the different materials are summarized in Table 1. Respective results are shown and discussed in Section 3.3.

### 2.6. Adhesion

**Figure 2:**
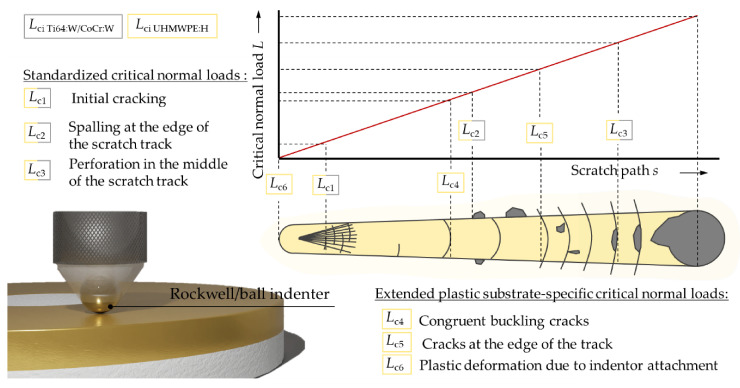
Principle and evaluation of the modified scratch test.

The coating adhesion was characterized by a standardized Rockwell-D penetration test (DuraJet 10G5, EMCO-TEST Prüfmaschinen, Kuchl, Austria) using a cone-shaped diamond indenter and digital light microscopy with ×100 magnification (DM4000 M, Leica Microsystems, Wetzlar, Germany), classifying into six adhesion strength classes HF1–HF6 depending on the occurring damage pattern [72,73,74,75]. Five indentations per specimen were evaluated for statistical purposes, whereby only the Ti64:W and CoCr:W coatings on the metallic substrates could be analyzed. To further quantify the adhesion for Ti64:W, CoCr:W and UHMWPE:H, Rockwell scratch tests according to DIN EN ISO 20502 [76] were performed (RTG-2, KTmfk, Erlangen, Germany). In addition, a ball indenter (100Cr6, radii *r* ≈ 1.4 and 2.0 mm) instead of a Rockwell indenter was used for modified scratch testing on UHMWPE:H following Sander et al. [77] due to better applicability to hard coatings on soft substrates. Thus, the failure events could be identified more clearly using digital light microscopy (DM4000 M, Leica Microsystems, Wetzlar, Germany). Similarly, modified scratch tests were performed for Ti64:W and CoCr:W using a ball indenter (WC, *r* ≈ 0.5 mm) as well. The principles followed for the assignment of error patterns to the standardized critical normal loads *L*_c1_ to *L*_c3_ [76] and the extended plastic substrate-specific critical normal loads *L*_c4_ to *L*_c6_ [77] are illustrated in Figure 2. Mean values were finally calculated from five repeated measurements for each scratch test type and specimen. Respective results are shown and discussed in Section 3.4.

## 3. Results and Discussion

In the following, the morphology and structure of the coating systems, the chemical and cytological characterization and the measured mechanical and adhesion properties are presented and discussed.

### 3.1. Coating Topography and Structure

**Figure 3:**
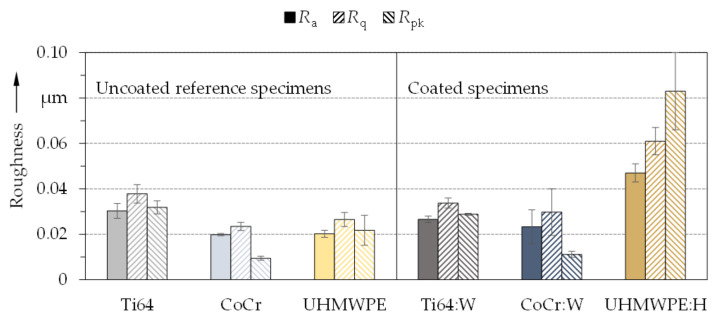
Average roughness values and standard deviation of the uncoated reference (left) and amorphous carbon coated (right) specimens (*n* = 5).

The average roughness values of the uncoated and coated surfaces of the metallic pins and the polymeric disks as used for the tribological experiments of part II [50] are summarized in Figure 3. Thereby, the values of uncoated reference specimens differed slightly by less than 0.04 µm. The uncoated Ti64 pins featured a higher roughness than the CoCr pins and the UHMWPE disks, whereby the values for the last two were comparable. Yet, the *R*_pk_ value of UHMWPE (≈ 0.022 µm) was about twice that of the CoCr pins (≈ 0.009 µm). The coated metallic specimens (Ti64:W, CoCr:W) revealed similar trends regarding the proportions between *R*_a_, *R*_q_ and *R*_pk_. Ti64:W exhibited slightly lower roughness values than uncoated Ti64, which could be attributed to a reduction of roughness grooves due to the sputtering technology. Thus, Ti64:W might be biotribologically more advantageous than bare Ti64. CoCr:W had the smoothest surface among the coatings. It was also smoother than Ti64 but rougher and more scattered than CoCr. Generally, the roughness values were within the typical range for metallic TKA femoral components [78] and also within the values specified by ISO 7207-2 [79] (*R*_a_ ≤ 0.1 µm). Especially UHMWPE:H displayed an increase of the *R*_pk_ value from approximately 0.022 µm to 0.083 µm and significantly higher roughness values compared to the other specimens including uncoated UHMWPE. This was due to a more complicated coating process. However, it should be emphasized that the roughness was still within typical values [80,81] and also well within limits for polymeric (*R*_a_ ≤ 2 µm) and metallic or ceramic (*R*_a_ ≤ 0.1 µm) tibial TKA inlays specified by ISO 7207-2 [79]. Therefore, the roughness of all uncoated and coated specimens can be considered representative and sufficiently low for cell growth [82,83].

**Figure 4:**
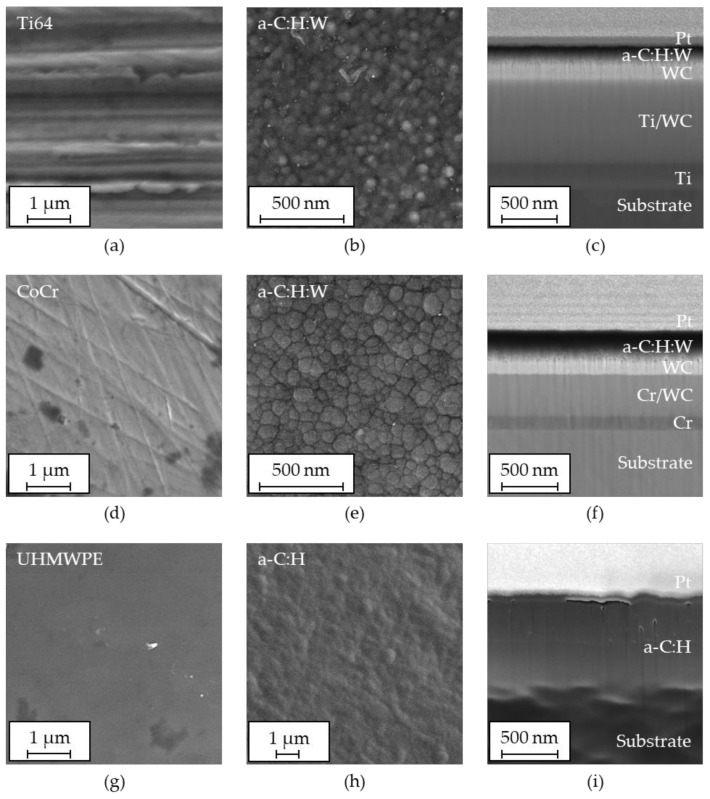
SEM top images of the uncoated polished substrate surfaces (left), the functional coating layers (middle) and FIB cross-sections (right) of Ti64:W (**a**–**c**), CoCr:W (**d**–**f**), and UHMWPE:H (**g**–**i**). The top layer (Pt) is not part of the original samples but originates from specimen preparation (protective layer).

The SEM images of uncoated and coated surfaces as well as FIB cross-sections revealing the coating architectures are shown in Figure 4. The plane view images on the left hand side show the representative morphology of the polished metallic pins (Figure 4a,d) as well as the UHMWPE disk (Figure 4g) prior to coating deposition. Although the surfaces were mechanically polished in a multi-stage process, some differences between the different materials can be observed, which account for the aforementioned roughness values. Thereby, the Ti64 pins featured more distinct polishing patterns than the CoCr pins and UHMWPE disks. This could be attributed to several material properties inherent to Ti64, such as its low Young’s modulus and low thermal conductivity, complicating surface finishing [84] and facilitating near-surface grain elongation and local plastic deformation [85] due to the sensitivity of the more ductile titanium *β* phase [86]. Furthermore, mechanically induced heat input can promote vanadium diffusion from the *β* to the more brittle *α* phase in near-surface regions [87,88]. Thus, the more inhomogeneous and rougher morphology of Ti64 (Figure 4a) results from locally different microstructures, surface hardness, built-up edges, heat-affected zones, and tensile residual stresses [84,89]. Despite similarities of the materials [90,91], the CoCr pins (Figure 4d) showed a smoother and more homogeneous surface due to its lower oxidation tendency [92] and the more easily machinable dominant face-centered cubic crystal structure [93] compared to the dominant hexagonal closed-packed crystal structure of Ti64. The UHMWPE could be polished comparatively easily and exhibited a homogenous and smooth surface with only occasional surface defects.

The images of the coated surfaces (Figure 4b,e,h) displayed slight cauliflower-like topographies indicating columnar growth [35]. This could be mainly attributed to the applied sputtering technology, which induced fewer defects than the droplet formation promoting arc evaporation. Thus, nano-roughness corresponding to the lateral column dimensions of about roughly 50 nm for Ti64:W (Figure 4b), 100 nm for CoCr:W (Figure 4e) and 125 nm for UHMWPE:H (Figure 4h) were observed. The columnar growth expanding towards the surface can also be observed in the FIB cross-sections (Figure 4c,f,i). The lack of contrast in the a-C:H:W functional layers of Ti64:W and CoCr:W is a consequence of their dense amorphous structure.

**Figure 5:**
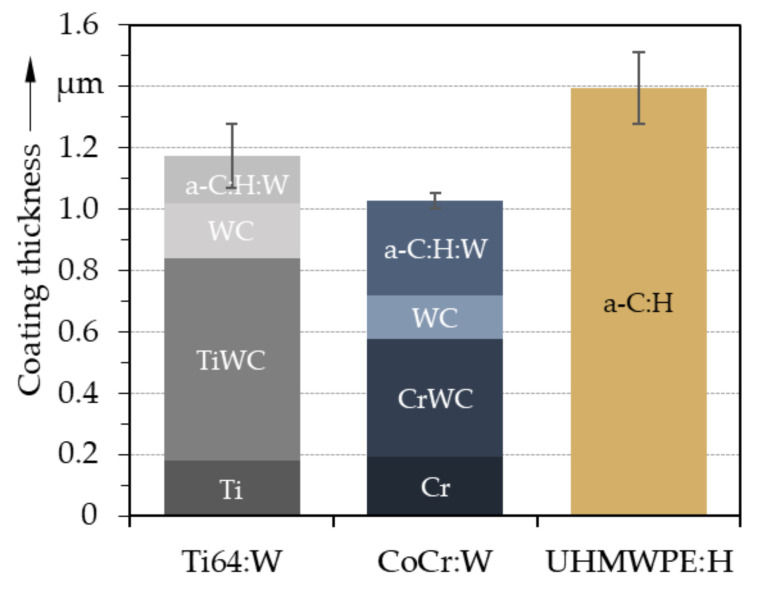
Averaged coating thicknesses and standard deviation (*n* = 5).

From both the FIB cross-sections and the averaged values measured from crater-grindings (see Figure 5), total coating thicknesses of around 1.2 ± 0.1 μm for Ti64:W, 1.0 ± 0.2 μm for CoCr:W and 1.4 ± 0.2 μm for UHMWPE:H were determined. Due to the multilayer structure of Ti64:W and CoCr:W, the actual a-C:H:W functional layers only had a thickness of roughly 0.2 µm and 0.3 µm, respectively. The adhesion layer was thinner for CoCr:W than for Ti64 due to differences in the growth rates within the coating process. Overall, however, these thicknesses as well as that of the monolayer UHMWPE:H coating were within the desired ranges, which represented a compromise between sufficient wear protection and keeping the maximum stresses under tribological loading within the substrate.

### 3.2. Chemical Properties and Cytological Interactions

**Figure 6:**
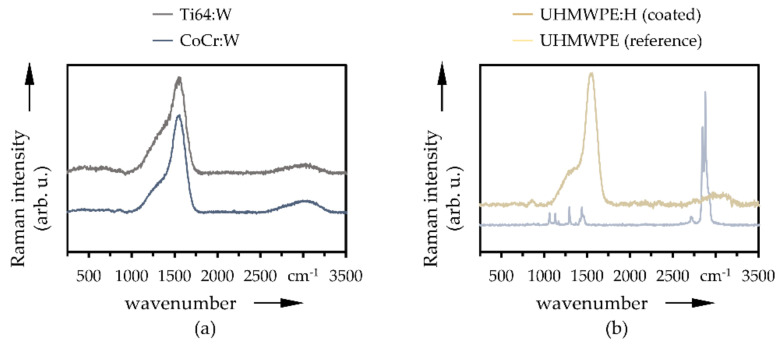
Averaged Raman spectra on the coated Ti64:W and CoCr:W disks and pins (**a**) as well as the reference and a-C:H-coated UHMWPE disk (**b**) (*n* = 3).

The averaged Raman spectra of the coated metallic Ti64:W and CoCr:W specimens (pins and disks) as well as the uncoated and coated UHMWPE disks are depicted in Figure 6. While the uncoated metallic specimens were not Raman active, the uncoated UHMWPE disks (Figure 6b, blue) featured characteristic intense peaks at 2848 and 2880 cm^−1^ as well as weaker peaks at 1065, 1293, 1305, 1435, and 2724 cm^−1^ [94,95,96,97]. Moreover, the Raman spectra of all DLC-coated specimens showed strong similarities with two pronounced peaks around 1360 and 1560 cm^−1^, which are typical positions for the D- and G-bands of amorphous carbon coatings [98]. A weakly pronounced peak around 850 cm^−1^ could also be observed, indicating trans-polyethyne [99,100,101]. Additionally, a broad and weak peak is visible between 2700 cm^−1^ and 3200 cm^−1^ [101] suggests a superposition of symmetric and antisymmetric stretching modes of hydrogen incorporation, such as CD_x_ and CH_x_ [98], due to 2D- and 2G-bands [102]. The intensity ratio of D- and G-band (*I*_D_/*I*_G_) yielded the content of sp^2^ hybridized carbon atoms, with a small value of the quotient describing a low sp^2^ content and a higher sp^3^ content, respectively [98]. Here, the *I*_D_/*I*_G_ ratios for Ti64:W, CoCr:W and UHMWPE:H can be approximated to be in the range to 0.2 and 0.3, which is characteristic of hydrogenated amorphous carbon coatings [101,103].

**Figure 7:**
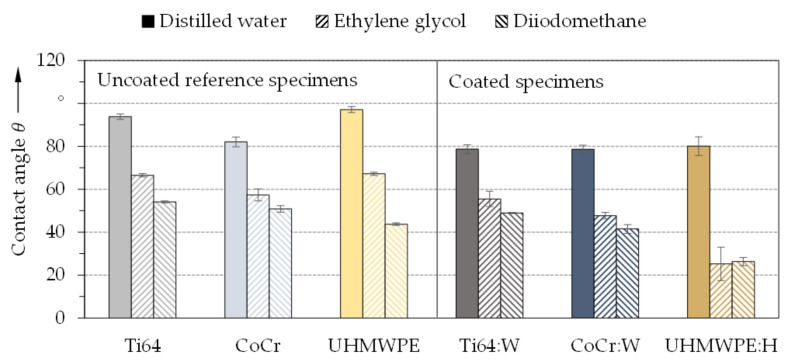
Averaged contact angles and standard deviation for the test liquids distilled water, ethylene glycol, and diiodomethane for the uncoated reference (**left**) and amorphous carbon coated (**right**) specimens (*n* = 5).

**Figure 8:**
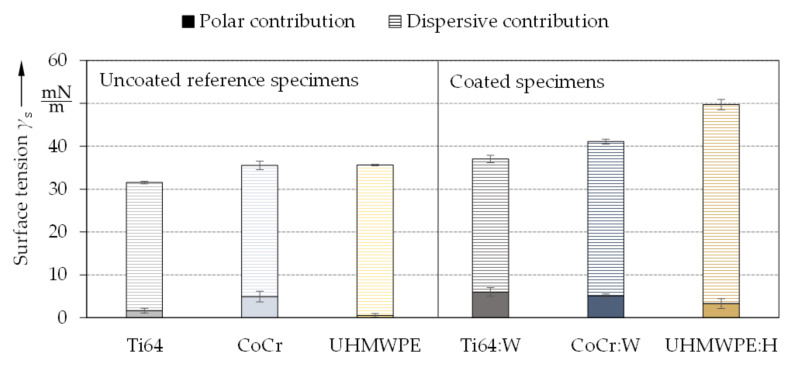
Average surface tension and standard deviation partitioned into the polar and dispersive contributions for the uncoated reference specimens and the amorphous carbon coated specimens (*n* = 5).

Furthermore, the contact angles and calculated surface tensions were analyzed to assess wettability and adhesion in order to predict the biological cell behavior [104]. The averaged contact angles *θ* and surface tensions *γ*_s_ of the uncoated reference specimens and the amorphous carbon coated specimens for the three tested liquids are shown in Figure 7 and Figure 8 respectively.

With distilled water, the contact angles of references CoCr and Ti64 were somewhat higher than reported by Hinüber et al. [105]. Since the surface topography substantially affects the wetting behavior [106], this could be attributed to the higher roughness of the specimens analyzed within this contribution. Moreover, the wettability of the uncoated reference substrates and the coated specimens demonstrated a noticeable difference. In this respect, the observed trends between uncoated and DLC-coated metallic and polymeric surfaces generally matched well the literature [107,108]. The lower contact angles and thus more hydrophilic character of coated UHMWPE:H compared to uncoated UHMWPE suggested better suitability for cell attachment [109] despite higher roughness [110]. The decrease in contact angles due to the a-C:H coating could be further traced back to polar functional groups on the surface, which enhance the interactions with polar liquids [111,112]. As pointed out by He et al. [47], improved wettability can be considered beneficial for biocompatibility and with regard to the biotribological behavior due to promoted adsorption of plasma proteins and synovial fluid cells, which may form a friction and wear-reducing film [81,111]. Possibly, more hydrophilic surfaces with more moderate water contact angles below 60° could be even more advantageous for cell-substrate compatibility [104,112,113,114].

A correlation between contact angles (Figure 7) and surface tension (Figure 8) was observed due to the polar contributions of the surface free energy [115]. Here, values between 31.6 ± 0.4 mN/m and 35.6 ± 0.3 mN/m were obtained for the uncoated substrates as well as between 37.0 ± 0.9 mN/m and 49.7 ± 1.5 mN/m for the amorphous carbon coatings. Thus, the surface tensions were considerably increased by the coatings, especially due to the higher polar contribution. This was in particular observable for Ti64:W as well as for UHMWPE:H and only slightly for CoCr:W when compared to the corresponding references, thus the coated surfaces can be considered more favorable for cell adhesion and interaction [116]. UHMWPE:H exhibited the smallest contact angles, the largest polar contribution (3.3 ± 1.2 mN/m) and thus the highest surface tension (49.7 mN/m). As reported by Valk et al. [117], adhesion of cells is maximized for polar contributions above 15 mN/m and minimized for values below 5 mN/m. Here, the polar contributions were significantly below 15 mN/m for all specimens, but above 5 mN/m for Ti64:W and CoCr:W as well as just slightly below for UHMWPE:H. Consequently, it can be expected that the adhesion of cells can be positively influenced by applying amorphous carbon coatings.

**Figure 9:**
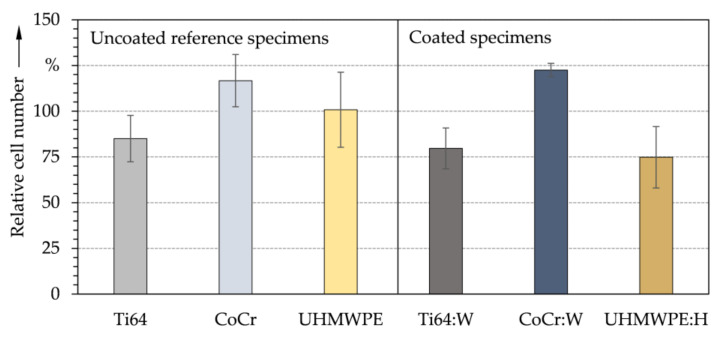
Average relative number of MG-63 cells and standard deviation for the uncoated reference (**left**) and amorphous carbon coated (**right**) specimens after indirect cell testing (*n* = 3).

As shown by indirect test method, MG-63 cells were able to survive and proliferate in the presence of the released material particles or substances from released particles (Figure 9). Besides components released by the specimens, there was a high osteoblastic cell layer formation on the bottoms of the well plates after 24 h, which was also reflected in the quantitative evaluation. The average relative cell numbers between tested specimens and positive controls did not reveal statistically significant differences (ANOVA). Thus, cytotoxic behavior in vitro could be excluded. Also, only the interaction of a monolayer of cells with the released substances was examined. When comparing the coated specimens to the positive controls as well as to the corresponding reference specimens, Ti64:W and UHMWPE:H had lower relative cell numbers. In contrast, CoCr:W had the highest relative cell number of all specimens, indicating favorable biocompatible behavior. Contrary to expectations based upon contact angles and surface tension, UHMWPE:H appears to have a rather low cell number compared to the other specimens. Therefore, it can be hypothesized that a metallic doping of the a-C:H coating with tungsten may have a positive effect on cell proliferation and cytocompatibility [118].

**Figure 10:**
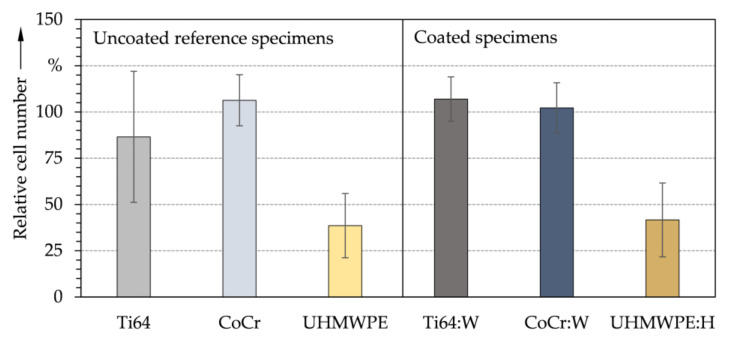
Average relative number of MG-63 cells and standard deviation for the uncoated reference (**left**) and amorphous carbon coated (**right**) specimens after direct cell testing (*n* = 3).

The interaction between the materials’ surfaces and the cells was further assessed by direct cell tests. Controls in which cells adhered and grew on the bottom of the well plate without the influence of any material were tested against direct adhesion and proliferation of cells on the specimens. The averaged relative cell numbers between tested specimens and positive controls after direct cell testing are depicted in Figure 10. Again, no statistically significant difference between uncoated and coated specimens could be observed. Compared to UHMWPE and UHMWPE:H, cell growth was particularly promoted on the metallic substrates (Ti64, CoCr, Ti64:W CoCr:W). Although CoCr:W and UHMWPE:H were rougher than the uncoated reference specimens, no increase in osteoblast proliferation was found. Furthermore, no substantially higher cell number was detected for UHMWPE:H despite featuring the highest surface tension. Yet again, this is consistent with the tendentially too low polar contribution [117]. While this result could be misinterpreted as critical biocompatibility, it is noteworthy that the inherent biocompatibility of UHMWPE is generally accepted [119]. The lower cell number on UHMWPE and UHMWPE:H can very likely be attributed to the different geometries of the specimens, which have an impact on the distribution and density of the cell layers. In this regard, complementary tests, such as water soluble tetrazolium test (WST) or 3-(4,5-dimethylthiazol-2-yl)-2,5-diphenyltetrazolium bromide test (MTT) [120], will be required to more accurately assess cell viability in the future.

**Figure 11:**
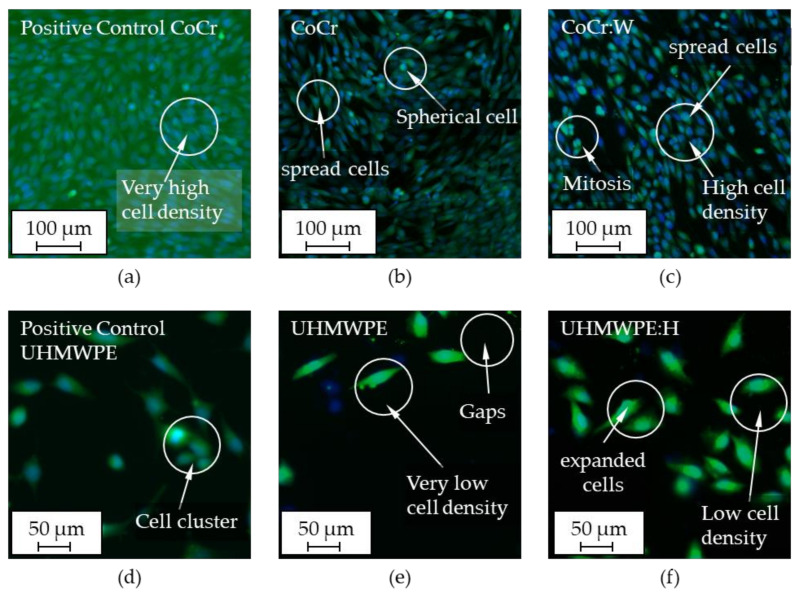
Representative fluorescence images of human osteoblastic cells of the positive control (**a**), uncoated (**b**) and coated CoCr (**c**) as well as the positive control (**d**), uncoated (**e**) and coated UHMWPE (**f**) after direct cell testing. The cell density on the positive controls was adjusted to the specimen geometry.

Fluorescence images of human osteoblastic cells after direct testing are illustrated in Figure 11. For positive controls and uncoated as well as coated CoCr and UHMWPE substrates, representative images for the highest and lowest cell counts from the direct measurements are shown. Initially, the osteoblastic cells adhered to the surfaces and spread out. Thereby, nuclei could be seen in blue color and vital cells in green color. To assess the cell morphology, a distinction was further made between round shapes, indicating weakly adherent or dead cells, as well as elongated and spread shapes, suggesting good cell adhesion, bioinert and bioactive behavior [105,121]. Regarding CoCr (Figure 11b) and compared to the positive control (Figure 11a), a high cell density with comparatively few cells stained by cytoplasmic calcein were observed. Occasionally, round cell shapes could be detected (Figure 11b), which indicated dead cells (blue) or non-adherent, vital cells (blue and green staining). For CoCr:W (Figure 11c), high cell density and clustering (accumulation of spread cells) could be seen as well. In general, cells had a round shape when they divided and subsequently unfolded when adhering the surface. The prevalence of stretched, accumulated and vital cells pointed towards good cell-surface adhesion, in turn favoring normal tissue-forming behavior in cell proliferation. In contrast, the cell density of positive control, UHMWPE as well as UHMWPE:H (Figure 11d–f) appeared to be substantially lower and less homogeneous compared to the metallic equivalents. Thereby, the cells showed a typical osteoblastic morphology, which could be clearly recognized by the calcein staining. UHMWPE:H (Figure 11e) featured a higher clustering of mainly live and expanded cells and thus an at least comparable or better biocompatibility compared to uncoated UHMWPE (Figure 11f).

Essentially, the studied amorphous carbon coatings can be classified as biocompatible based upon our short-term indirect and direct cell tests since no negative influences were observed. Released trace elements from the metallic surfaces, such as Co and Cr, can have a negative impact on cytocompatibility and promote toxic tissue reactions when concentrations are exceeding certain toxicity limits in the human body [122]. Normally, Ti64 and CoCr are well protected by the formation of thin passive layers when in contact with oxygen, which reduces metallic ion release. Following tribological stressing in knee replacements, however, this protecting layer may be removed partially or entirely. As stated by Mansano et al. [118], amorphous carbon coatings are able to form a diffusion barrier that helps to reduce the release of metallic ions or particles. Further experiments on stress tested specimens will be required in future studies to evaluate the long-term behavior of the amorphous carbon coatings.

### 3.3. Mechanical Properties

**Figure 12:**
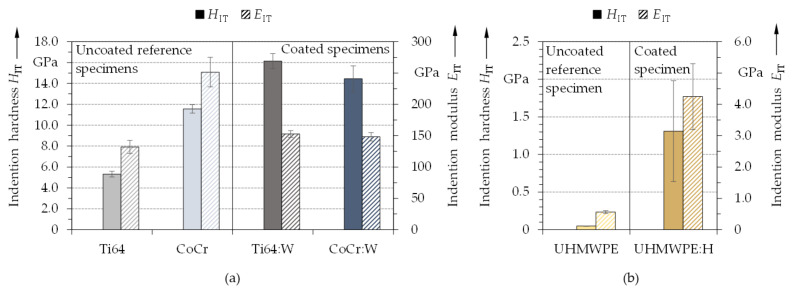
Average indentation hardness and modulus with standard deviation for the metallic substrates (left) as well as Ti64:W and CoCr:W (right) (**a**) and the polymeric substrate (left) as well as UHMWPE:H (right) (**b**) (*n* = 10).

The average values for the indentation hardness and modulus are shown in Figure 12, whereas calculated *H*_IT_/*E*_IT_ and the *H*_IT_^3^/*E*_IT_^2^ ratios are summarized in Table 2. Apparently, hardness and elasticity differed not only between the uncoated and coated groups, but also between the various substrates and coatings. With an *H*_IT_ of about 5.3 GPa and an *E*_IT_ of 131.9 GPa, Ti64 exhibits roughly half the values of CoCr (*H*_IT_ ≈ 11.6 GPa and *E*_IT_ ≈ 251.5 GPa). Through the applied a-C:H:W coating (Figure 12a), the hardness was in-creased to roughly 16.1 GPa for Ti64:W and 14.4 GPa for CoCr:W, which corresponded to a threefold and quarter rise, respectively. At the same time, the indentation modulus only slightly increased to 152.8 GPa for Ti64:W and even decreased to about 148.3 GPa for CoCr:W. Accordingly, the *H*_IT_/*E*_IT_ and *H*_IT_^3^/*E*_IT_^2^ ratio were also substantially increased by the coatings (Table 2). Similarly, the a-C:H coating on the polymeric substrate resulted in an increase in indentation hardness and modulus as well, although the values were an order of magnitude lower than for the metallic substrates (Figure 12b). Thus, *H*_IT_ and *E*_IT_ values of roughly 47.6 MPa and 0.56 GPa for UHMWPE as well as 1.3 GPa and 4.3 GPa for UHMWPE:H were measured, which represented an increase by a factors of circa 27 and 7.5, respectively. This was also reflected in the distinct increase of *H*_IT_/*E*_IT_ and *H*_IT_^3^/*E*_IT_^2^ (Table 2). The latter can be seen as an indicator for the material’s capability to undergo deformation while withstanding mechanical and tribological stresses [31,35]. Here, Ti64 was found to exhibit lower ratios than CoCr. UHMWPE showed a lower *H*_IT_^3^/*E*_IT_^2^ value than Ti64 and CoCr, while the *H*_IT_/*E*_IT_ ratio was comparatively high. In all cases, the ratios were significantly enhanced by the coating. It can be expected that the higher coating hardness is able to protect the substrates from abrasive and adhesive wear as well to shift crack formation towards higher stresses. Simultaneously, the relatively smaller indentation modulus might contribute to an increased ability of the coating system to sag without flowing (elastic strain to failure) [123]. Thus, the pressures from tribological loading can be decreased by increasing the contact dimensions [35]. When comparing the *H*_IT_^x^/*E*_IT_^y^ ratios to values published in literature [34,124,125], the coatings studied within this contribution were found to be in the upper range for Ti64:W and CoCr:W. For UHMWPE:H, the values even exceeded those from comparable studies in literature [47,111,126]. Therefore, it can be assumed that the developed a-C:H:W and a-C:H coatings will exhibit a highly advantageous wear behavior [42], which is also substantiated by the biotribological investigations in part II [50].

### 3.4. Adhesion

**Figure 13:**
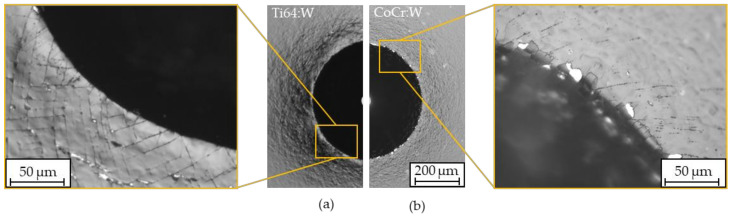
Representative Rockwell-D indentations for Ti64:W (**a**) and CoCr:W (**b**) (*n* = 5).

Representative light microscopic images after standardized Rockwell-D penetration testing are depicted in Figure 13. Thereby, Ti64:W showed a good adhesion strength of HF2 while CoCr:W exhibited a good to satisfactory adhesion strength and was ranked as HF2—3. Furthermore, a slight sink-in around the indentation could be observed, which was more pronounced for Ti64:W than for CoCr:W due to the low substrate hardness. In principle, the coatings’ adhesion therefore met the industrial standard requirements (better than HF4) and can be regarded as sufficient.

**Figure 14:**
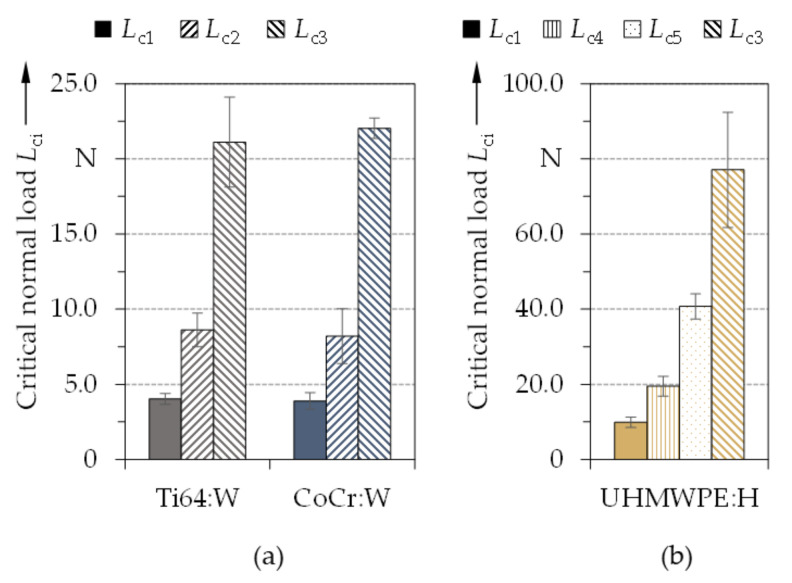
Average values of critical normal loads *L*_ci_ and standard deviation for Rockwell indentation on Ti64:W and CoCr:W (**a**) and for 100Cr6 ball indentation (*r* ≈ 2 mm) on UHMWPE:H (**b**) (*n* = 5).

The average critical normal loads for Ti64:W and CoCr:W determined with a Rockwell indenter and for UHMWPE:H generated with a 100Cr6 spherical indenter (*r* ≈ 2 mm) are illustrated in Figure 14a,b. Thereby, Ti64:W and CoCr:W exhibited similar normal loads, which indicated good adhesion and also matched well the previous Rockwell indentations results. Therefore, the a-C:H:W coatings studied within this contribution featured adhesion properties comparable to the amorphous carbon coatings from Escudeiro et al. [34] on Ti64 as well as from Döring et al. [16] on CoCr. Moreover, higher *L*_ci_ values were derived in comparison to the DLC coatings from Dorner et al. [125] on Ti64 or from Tremmel et al. [127] on CoCr. Since there is a nearly linear relationship between the critical normal load and the indenter radius [128,129,130], critical failure events were shifted towards higher values by increasing the contact radius using the WC ball indenter, see Table 3. Thus, a better comparability with the values for UHMWPE:H was to be attained.

**Figure 15:**
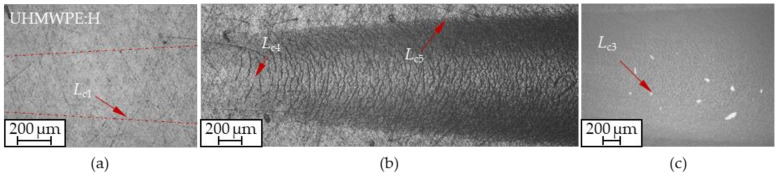
Representative scratch track for the determination of critical normal loads *L*_c1_ (**a**), *L*_c4_ and *L*_c5_ (**b**) as well as *L*_c3_ (**c**) on UHMWPE:H generated by a 100Cr6 ball indenter (*n* = 5).

A representative scratch track on UHMWPE:H with characteristic damage phenomena is pictured in Figure 15. The first cracks at the edge of the scratch track (Figure 15a) can be distinguished from congruent buckling cracks (Figure 15b) and cracks at the edge of the track (Figure 15c). Other damage phenomena, such as spalling at the edge of the scratch track (*L*_c2_), did not occur for UHMWPE:H, indicating good coating cohesion. A high agreement of the results was mainly achieved by the spherical indenter with a large radius, which favored separation of failure events and allowed to investigate the critical adhesive coating failure (*L*_c3_) after all [77]. Although UHMWPE:H exhibited considerably higher critical normal loads than Ti64:W and CoCr:W, the coated metallic specimens can hardly be directly compared to UHMWPE:H due to influences from the substrates and the coating architecture. A comparison of the values measured on UHMWPE:H with a Rockwell indenter, see Table 3, was also not feasible due to the proximity of critical damage values and because the small indenter radius (*r* = 200 µm) stressed the substrate in particular instead of the coating-substrate interface [77]. This was also confirmed by the near initial deformation of the polymer substrate at 1 N (*L*_c6_ value in Table 3). When comparing the Hertzian pressures for the pairings between Ti64, CoCr as well as UHMWPE and the Rockwell diamond (*p*_H,Ti64,Lc1_ = 6715 MPa, *p*_H,CoCr,Lc1_ = 9553 MPa and *p*_H,UHMWPE,Lc1_ = 213 MPa), it becomes evident that this fairly exceeded the tensile strength of cortical bone as well as the tensile strength of the substrates [131]. Likewise, this clearly surpasses the pressures to be expected in TKAs [81,132]. Overall, the adhesion of the investigated coatings to all substrates can therefore be considered as sufficient for the biotribological requirements in knee endoprostheses.

## 4. Conclusions

This contribution investigated cytocompatibility, chemical, mechanical and adhesion properties of physical vapor deposited a-C:H:W coatings on Ti64 and CoCr as well as an a-C:H coating on UHMWPE. Roughness and coating thickness measurements, FIB-SEM, Raman spectroscopies, contact angle measurements, indirect and direct cell testing, indentation hardness and elasticity measurements, Rockwell and scratch tests were employed for this purpose. For the studied materials and coatings, the following conclusions could be drawn:

▪ The deposited coatings displayed a morphology as well as composition typical for amorphous carbon coatings. The roughness of the coatings was higher than that of the substrates, especially for UHMWPE.
▪ Initial screening with contact angles and surface tensions as well as indirect and direct in vitro biocompatibility studies on DLC coatings comparable to the substrates showed no cytotoxic effects of the surface treatment and confirmed the approach of the biomedical application.
▪ The developed coatings featured excellent mechanical properties with a substantial enhancement of *H*_IT_^x^/*E*_IT_^y^ ratios, indicating favorable biotribological wear behavior.
▪ The adhesion of the coatings to the Ti64, CoCr and UHMWPE substrates can be considered as sufficient for the usage in total knee replacements.
▪ It can be assumed that the amorphous carbon coatings presented in this contribution are able to outperform uncoated metallic-polymeric reference pairings under biotribological stresses. This will be further elaborated in part II of this study [50].

## Figures and Tables

**Table 1:** Nanoindentation parameters and settings.

Parameters	Ti64	Ti64:W	CoCr	CoCr:W	UHMWPE	UHMWPE:H
Maximum load	0.5 mN	0.5 mN	0.5 mN	0.5 mN	0.05 mN	0.05 mN
Application time	15 s	15 s	4 s	4 s	3 s	3 s
Delay time after lowering	-	-	-	-	30 s	30 s
Poisson’s ratio	0.3	0.3	0.3	0.3	0.46	0.3

**Table 2:** Averaged values and standard deviation of the *H*_IT_/*E*_IT_ and *H*_IT_^3^/*E*_IT_^2^ ratios for uncoated amorphous carbon coated specimens (*n* = 10).

Designation	*H*_IT_/*E*_IT_	*H*_IT_^3^/*E*_IT_^2^
Ti64	0.040 ± 0.003	0.009 ± 0.002 GPa
CoCr	0.046 ± 0.005	0.025 ± 0.006 GPa
UHMWPE	0.085 ± 0.009	0.001 ± 0.001 GPa
Ti64:W	0.106 ± 0.006	0.180 ± 0.027 GPa
CoCr:W	0.097 ± 0.010	0.137 ± 0.038 GPa
UHMWPE:H	0.309 ± 0.175	0.125 ${\pm \;}_{0.125}^{0201}$ GPa

**Table 3:** Average critical normal loads *L*_ci_ and standard deviation for Ti64:W (*n* = 3), CoCr:W (*n* = 5) and UHMWPE:H (*n* = 5). Values marked with “-” were not measured.

Coating	Indenter	*L*_c1_/N	*L*_c2_/N	*L*_c3_/N	*L*_c4_/N	*L*_c5_/N	*L*_c6_/N
Ti64:W	Ball, *r* ≈ 0.5 mm	2.9 ± 1.7	24.2 ± 8.4	37.8 ± 4.0	-	-	-
CoCr:W	Ball, *r* ≈ 0.5 mm	5.0 ± 0.4	38.9 ± 4.3	52.4 ± 2.3	-	-	-
UHMWPE:H	Rockwell	2.9 ± 0.3	-	-	5.9 ± 0.8	10.6 ± 0.6	1.0 ± 0.7

## Data Availability

The data presented in this study are available on request from the corresponding author.

## Nomenclature

*E*_IT_	Indentation modulus
*f*	Pulse frequency
*HF*	Adhesion strength class
*H*_IT_	Indentation hardness
*I*_D_	D-band Raman intensity
*I*_G_	G-band Raman intensity
*L*_c,i_	Critical normal load
*p*_H,i_	Maximum Hertzian contact pressure
*R*_a_	Average mean roughness
*R*_pk_	Reduced peak height
*R*_q_	Root-mean-squared roughness
*RRT*	Reverse recovery time
*s*	Scratch path
*γ*_s_	Surface tension
*θ*	Contact angle
*ν*	Poisson’s ratio
